# Public Information Demand During the 2026 Bundibugyo Virus Outbreak: A Dual Infodemiology Analysis Across Six Ebola Virus Disease Outbreaks

**DOI:** 10.3390/v18090987

**Published:** 2026-09-08

**Authors:** Hakkı Öztürk, Berrak Itır Aylı

**Affiliations:** 1 Afyonkarahisar Provincial Health Services Directorate, Afyonkarahisar 03200, Türkiye; 2 Faculty of Life Sciences, University of Westminster, London W1B 2HW, UK

**Keywords:** Bundibugyo ebolavirus, Central Africa, Ebola virus, disease outbreaks, Google Autocomplete, Google Trends, infodemic, misinformation

## Abstract

The 2026 Bundibugyo virus disease outbreak in the Democratic Republic of the Congo and Uganda, the first Ebola-related PHEIC caused by a species lacking a licensed vaccine, has been accompanied by an information crisis marked by conspiracy-driven violence against response teams and destruction of treatment centres. Understanding what populations actually seek to know during such events is essential for designing effective communication strategies, yet the content of public information demand during Ebola outbreaks has not been systematically characterised. We combined Google Autocomplete query extraction (7347 unique queries from 83 seed terms in English and French) with Google Trends temporal analysis across six Ebola outbreaks (2014–2026). The autocomplete suggestion landscape was dominated by risk quantification and transmission concerns. Google Trends temporal analysis showed moderate to strong co-movement between misinformation-related search terms and overall Ebola search interest (Spearman ρ = 0.359–0.697, nominal *p* < 0.001). Substantial differences emerged between the English-language and French-language suggestion sets: French-language suggestions contained six times more misinformation and conspiracy content (4.9% vs. 0.8%) and nearly nine times more fear-related content (3.5% vs. 0.4%) than English-language suggestions. These findings demonstrate that infodemic management strategies designed in Anglophone contexts are inadequate for the populations most at risk and should inform linguistically adapted interventions for the ongoing response.

## 1. Introduction

Ebola virus disease (EVD) is a severe and often fatal zoonotic illness caused by viruses of the genus *Orthoebolavirus* within the family *Filoviridae*. First identified in 1976 following simultaneous outbreaks in Nzara, Sudan, and Yambuku, Democratic Republic of the Congo (DRC), EVD has since caused sporadic but devastating epidemics across sub-Saharan Africa [1,2]. Three species, Zaire ebolavirus, Sudan ebolavirus, and Bundibugyo ebolavirus, are responsible for the majority of human disease, with case fatality rates ranging from 25% to 90% depending on the species, outbreak setting, and availability of medical care [1,3]. The 2014–2016 West Africa epidemic, caused by Zaire ebolavirus, remains the largest on record, with over 28,600 cases and 11,300 deaths across Guinea, Liberia, and Sierra Leone, and secondary transmission events in Nigeria, Senegal, Mali, the United States, Spain, Italy, and the United Kingdom [4,5].

Since the West Africa epidemic, EVD outbreaks have continued to recur, particularly in the DRC, which has experienced 17 documented outbreaks since 1976 [6]. The 2018–2020 North Kivu/Ituri outbreak was the country’s largest, with 3481 cases and 2299 deaths, compounded by armed conflict and community resistance to response activities [7,8]. Uganda experienced a Sudan virus outbreak in 2022 (164 cases, 77 deaths) and a smaller outbreak in early 2025 [9]. In May 2026, a new outbreak of Bundibugyo virus disease (BVD) was declared in Ituri Province, DRC, rapidly spreading to neighbouring Uganda and marking only the third known outbreak of this viral species [10]. The World Health Organization (WHO) declared the outbreak a Public Health Emergency of International Concern (PHEIC) on 17 May 2026, and the Africa Centres for Disease Control and Prevention declared a Public Health Emergency of Continental Security the following day [11]. As of July 2026, over 3300 cases have been identified and over 1400 deaths reported across multiple provinces, making it the third-largest and fastest-growing Ebola outbreak on record [10,11,12]. Critically, there is no licensed vaccine or specific therapeutic agent approved for Bundibugyo virus, distinguishing this outbreak from recent Zaire ebolavirus events where the Ervebo vaccine was available for ring vaccination [10,13].

EVD outbreaks have historically been accompanied by a parallel crisis of information, which the WHO has termed an “infodemic”, defined as an overabundance of information, including false or misleading content, that occurs during disease outbreaks and makes it difficult for people to identify trustworthy sources and adopt protective behaviours [14,15]. During the 2014–2016 epidemic, misinformation about EVD transmission, prevention, and treatment proliferated through both digital and traditional media channels, contributing to community mistrust, resistance to public health interventions, and went as far as targeted violence against healthcare workers [16,17]. In the DRC, where successive Ebola outbreaks have intersected with armed conflict, poverty, and deep-rooted mistrust of government and international actors, misinformation has fuelled extreme acts such as attacks on Ebola treatment units, refusal of safe burial practices, and flight of suspected patients from isolation facilities [18,19]. A rapid review of misinformation during large-scale infectious disease outbreaks since 2000 revealed how widespread Ebola misinformation was by documenting that up to 68% of individuals believed the disease could spread through mere touch, 75% lacked comprehensive knowledge, and nearly 46% believed in at least one conspiracy theory [20]. The 2026 Bundibugyo outbreak shows the results of these misinformation trends, with documented attacks on burial teams, destruction of Ebola treatment centres, and the rapid circulation of conspiracy theories linking the outbreak to political motives and international exploitation [19,21].

These challenges underscore the importance of understanding not only the epidemiology of Ebola outbreaks but also the dynamics of public information demand and misinformation, highlighting the need for dedicated surveillance approaches.

Infodemiology, defined by Eysenbach as “the science of distribution and determinants of information in an electronic medium, specifically the Internet, or in a population, with the ultimate aim to inform public health and public policy” [22], offers a systematic framework for studying public information-seeking behaviour during health crises. A related concept, infoveillance, involves the continuous monitoring of online health information for surveillance purposes [23]. Within this paradigm, Internet search data, particularly data from Google, which commands over 90% of the global search engine market, serve as a naturalistic proxy for the health information needs, concerns, and misconceptions of the general public [24,25]. Two Google-based tools have emerged as widely used instruments in infodemiology research: Google Trends, which provides normalised relative search volume (RSV) data over time and across geographies [26], and Google Autocomplete (also known as Google Suggest), which returns algorithmically generated query suggestions based on aggregated user search behaviour [27].

The application of these tools to Ebola has been limited. Alicino et al. (2015) conducted the only dedicated Google Trends analysis of Ebola, examining RSV patterns during the 2014–2016 West Africa epidemic and correlating search intensity with weekly case counts and the Human Development Index [28]. Their study demonstrated a strong global correlation between Ebola case incidence and search volume but also highlighted the distorting influence of media coverage and the digital divide on search data in affected countries. Towers et al. (2015) focused mainly on Twitter data to model the “contagion of fear” during the United States Ebola transmission events of 2014, conceptualising information-seeking as an epidemic process with its own recovery dynamics [29]. Further studies were conducted on social media data; Oyeyemi et al. (2014) and Sell et al. (2020) analysed Ebola-related misinformation on Twitter, identifying prevalent themes including false transmission claims, conspiracy theories, and unverified cures [16,17].

However, although Google Trends has been applied in infectious disease infodemiology, with several studies examining search behaviour during the COVID-19 outbreak, the use of Google Autocomplete as a complementary tool for health-related research remains comparatively limited [28,30,31,32]. Unlike Google Trends, which quantifies the volume of search activity over time, Google Autocomplete captures the content of that activity: the specific questions, concerns, and phrasings that users bring to the search engine [27]. A small but growing body of literature has demonstrated the value of this distinction. One study used autocomplete predictions to document the real-time propagation of COVID-19 conspiracy theories through Google’s suggestion algorithm, showing that misinformation narratives were actively surfaced to users before they had finished typing their queries [33]. Valera et al. [30] applied a similar approach across English and Spanish during the early months of the COVID-19 pandemic, revealing significant language-based differences in the thematic content of autocomplete suggestions. However, no study has applied Google Autocomplete to Ebola virus disease, and to our knowledge, the method has not previously been combined with Google Trends temporal analysis in the context of any acute infectious disease outbreak; a gap that limits our understanding of both what populations seek to know during epidemics and how those information needs evolve over time.

Despite the recognised severity of Ebola-related infodemics and the availability of Internet-based surveillance tools, several critical questions remain unanswered. No study has (i) applied Google Autocomplete to systematically extract and thematically categorise Ebola-related public queries, (ii) compared autocomplete content patterns across the multiple Ebola outbreaks of the past decade, (iii) examined these patterns in both English and French to capture language-specific differences in the suggestion landscape or (iv) analysed the temporal relationship between outbreak events and misinformation-related search terms. The ongoing 2026 Bundibugyo virus outbreak, the first PHEIC-designated Ebola event caused by a species for which no licensed vaccine exists, provides both urgency and a unique analytical opportunity, as the public’s search behaviour during this novel-species outbreak may differ qualitatively from prior Zaire or Sudan ebolavirus events.

This study therefore aimed to characterise the online public information landscape related to EVD using a dual infodemiology approach combining Google Autocomplete query extraction with Google Trends temporal analysis. Specifically, the objectives were to (i) identify and thematically categorise the autocomplete queries generated in response to Ebola-related seed terms in English and French, (ii) compare the thematic distribution of queries between languages, (iii) map the temporal dynamics of Ebola-related search interest across six outbreak periods from 2014 to 2026, (iv) examine the temporal correlation between overall Ebola search interest and misinformation-related queries, and (v) identify geographic patterns in Ebola-related search interest. These five objectives constitute components of a single descriptive characterisation of the public information landscape during Ebola outbreaks, rather than independent analytical investigations; they are reported together because the integration of content, temporal, and geographic dimensions provides a more complete portrait than any single dimension alone.

## 2. Materials and Methods

### 2.1. Study Design

This cross-sectional infodemiology study employed a dual-method approach combining Google Autocomplete query extraction and Google Trends temporal analysis to examine the online public information landscape related to Ebola virus disease (EVD). The study framework was informed by Eysenbach’s infodemiology and infoveillance paradigm [22,23], which leverages Internet-based data sources to track health information demand and supply patterns at the population level. Data collection was conducted in July 2026, during the ongoing Bundibugyo virus disease (BVD) outbreak in the Democratic Republic of the Congo (DRC) and Uganda, which was declared a Public Health Emergency of International Concern (PHEIC) by the World Health Organization (WHO) on 17 May 2026 [11]. The study analysed autocomplete content and search interest patterns across six major Ebola outbreaks spanning 2013–2026, in two languages (English and French), to capture language-specific differences in the suggestion landscape.

The two methods address distinct but complementary dimensions of public information demand: Google Autocomplete captures the content of the suggestion landscape at a single time point, while Google Trends captures the temporal dynamics of aggregate search volume over time. These methods are not intended to validate one another; rather, the autocomplete analysis identifies what users are presented with when searching, while the Trends analysis shows when and how intensely such searching occurs. The integration is descriptive: by mapping both the ‘what’ and the ‘when’ of Ebola-related search activity, the study provides a more complete characterisation than either method alone, while acknowledging that each component has distinct limitations and should be interpreted on its own terms.

As the study relied exclusively on publicly available, anonymised, and aggregated Internet search data with no involvement of human participants, ethical approval was not required.

### 2.2. Data Sources

Google Autocomplete (also referred to as Google Suggest) is a predictive search feature that generates real-time query suggestions as users type into the Google search bar. These suggestions are algorithmically derived from aggregated search behaviour across Google’s user base, reflecting frequently entered and trending queries [27]. Unlike curated datasets, autocomplete suggestions offer a naturalistic proxy for the questions and concerns that the general public brings to search engines, making them an important tool for infodemiology research. We accessed autocomplete suggestions programmatically using a Python script executed in Google Colaboratory (Google LLC, Mountain View, CA, USA). The script queried Google’s publicly available autocomplete suggestion service (via HTTP GET requests using the Python requests library (version 2.31), which returns up to ten suggestions per query in JSON format.

Google Trends is a free, publicly accessible tool provided by Google that reports the relative search volume (RSV) of user-specified search terms over defined time periods and geographies [32]. RSV values are normalised on a 0–100 scale relative to the highest point of search interest within the selected parameters, enabling temporal and cross-regional comparisons of search intensity. Google Trends data were accessed via the pytrends Python library (version 4.9.3), which interfaces with the Google Trends API.

### 2.3. Autocomplete Data Collection

A comprehensive set of seed terms was developed to capture the breadth of Ebola-related autocomplete content. Seed terms were generated through (i) a review of published Ebola infodemiology literature, (ii) consultation of WHO and CDC Ebola fact sheets and frequently asked questions pages, (iii) a preliminary exploration of Google Autocomplete and Google Trends related queries for “Ebola”, and (iv) expert consensus among the study authors. The final seed term list comprised 47 English and 36 French terms spanning ten conceptual domains: core disease terminology, transmission and contagion, symptoms and clinical presentation, treatment and medical care, vaccination, prevention and protection, epidemiology and outbreak updates, travel and risk, psychosocial concerns, and the 2026 Bundibugyo-specific context. The complete list of seed terms is provided in Supplementary Table S1.

However, it is important to note that English and French seed term lists were developed independently to reflect the terminology and phrasing conventions of each language, rather than as direct translations. The French list comprised 36 terms (compared to 47 in English) because several English terms lacked natural French equivalents or produced no autocomplete output in preliminary testing. This asymmetry means that the two suggestion sets are not directly comparable in terms of their generative inputs, and language-based differences in the thematic distribution of suggestions may partly reflect differences in the seed terms used rather than differences in underlying search behaviour. We report these comparisons descriptively and caution against interpreting them as evidence of differences between linguistically defined populations.

To maximise the breadth of captured queries, each seed term was systematically expanded using an alphabetical suffix technique, whereby each letter of the Latin alphabet (a–z) was appended to the seed term (e.g., “ebola a”, “ebola b”, … “ebola z”). This approach exploits the autocomplete algorithm’s sensitivity to partial input, generating a wider array of suggestions than the seed term alone. For each seed term and its 26 alphabetical variants, up to 10 autocomplete suggestions were retrieved, yielding a theoretical maximum of 270 queries per seed term (10 × 27 inputs).

All queries were collected on 28 July 2026 using a standardised protocol. To minimise the influence of personalisation on autocomplete results, queries were submitted (i) without user authentication (no Google account login), (ii) from a single IP address via a virtual private network (VPN) routed through a server located in Türkiye, and (iii) with browser cookies and search history cleared prior to each session. English-language queries were collected with the language parameter set to *hl* = *en* and no geographic restriction (global scope). French-language queries were collected with *hl* = *fr*, also without geographic restriction. However, it is essential to note that these parameters used in the autocomplete API specify the interface language, not the geographic origin of the suggestions. Suggestions generated with *hl* = *fr* may reflect French-language search patterns globally, including users in France, Belgium, Canada, and West Africa, rather than specifically Francophone African populations affected by Ebola outbreaks. The language-based comparisons should therefore be understood as describing differences between French-language and English-language autocomplete ecosystems worldwide, not as a direct comparison between Anglophone and Francophone African communities. An automated rate-limiting delay of 0.3–1.5 s was imposed between successive API requests to avoid server-side throttling. Duplicate suggestions were removed at the point of collection using a case-insensitive deduplication algorithm.

Given the dynamic nature of autocomplete algorithms, which may vary outputs based on temporal trends, geographic signals, and algorithmic updates, the following reproducibility measures were implemented: (i) all data were collected within a single 8 h window to minimise temporal drift; (ii) raw and classified datasets were archived for independent verification. We acknowledge that autocomplete outputs are inherently non-static and that replication at a later date may yield partially different results, reflecting changes in public search behaviour rather than methodological inconsistency.

An important methodological consideration is that Google Autocomplete suggestions are not a direct, unmediated record of individual search queries; they are algorithmically generated predictions that incorporate query popularity, trending topics, geographic signals, and content policies [27]. We therefore do not claim that autocomplete suggestions represent a one-to-one mapping of public information needs. Rather, they capture the information landscape that users encounter when they begin searching; the set of queries that Google’s algorithm deems most relevant or popular for a given input. This distinction is itself infodemiologically significant: whether a suggestion reflects a frequently typed query or an algorithmically amplified one, its functional impact is the same, as it is presented to every user who begins typing the relevant seed term. Autocomplete thus serves as a de facto curatorial layer between user intent and information access [33], and its content shapes information-seeking trajectories regardless of how individual suggestions were generated.

### 2.4. Thematic Categorisation

Collected autocomplete queries were classified into thematic categories using a priority-based keyword-matching algorithm. The classification framework was developed *a priori* based on (i) the WHO infodemic management framework, (ii) published literature on Ebola-related misinformation, (iii) clinical domains relevant to EVD, and (iv) emergent themes identified during preliminary data exploration. Twelve mutually exclusive categories were defined (Table 1):

Each query was processed through the classification algorithm in a fixed priority order, and assigned to the first category for which a keyword match was identified. This priority-based approach was adopted to resolve cases where a single query could match multiple categories (e.g., “ebola natural cure conspiracy” matches both Misinformation and Traditional Remedies); in such cases, the higher-priority category was assigned. The priority order was determined by the specificity of the category, with narrower, more distinctive categories (e.g., Misinformation & Conspiracy) taking precedence over broader ones (e.g., Epidemiology & Outbreak Updates). Queries that did not match any category were classified as “Other/Unclassified” but were subjected to a secondary descriptive sub-classification to characterise their content. Six post hoc sub-categories were identified through manual inspection: Geographic/Location variants, Definition/Education queries, Health System/Policy queries, Media/Entertainment references, Science/Biology queries, and Epidemiological Metrics queries. Queries that could not be assigned to any sub-category were labelled ‘Truly Ambiguous’.

To assess the reliability of the automated classification, a random 10% sample of all classified queries (stratified by language) was independently re-classified by a second reviewer blinded to the algorithm’s output. Inter-rater agreement was quantified using Cohen’s kappa (κ = 0.91) and revealed excellent agreement. Discrepancies were resolved by consensus discussion between the authors and the reviewer. This aggregate measure may not fully capture classification reliability for low-frequency categories (e.g., Misinformation & Conspiracy, Traditional & Alternative Remedies), where the absolute number of validated queries in the 10% sample is small. Category-specific agreement statistics were not computed due to the limited number of observations per category in the validation sample. The reliability of conclusions drawn from these smaller categories should therefore be interpreted with appropriate caution.

To address this limitation, both authors independently conducted a complete manual review of all queries assigned to the three lowest-frequency substantive categories: Misinformation & Conspiracy (*n* = 204), Psychosocial & Fear (*n* = 136), and Traditional & Alternative Remedies (*n* = 14). Any queries that did not match their domain were reclassified and disagreements were resolved by consensus; no category showed a mismatch rate above 5%. This post hoc verification provides direct evidence that the automated classification performed adequately for the categories most central to the study’s interpretive claims.

### 2.5. Google Trends Temporal Analysis

Google Trends data were retrieved to examine the temporal dynamics of Ebola-related search interest across multiple outbreak periods. The following analyses were conducted:

- Long-term trend analysis. Monthly RSV data for the search term “Ebola” were retrieved globally for the period from 1 January 2013 to 29 July 2026. This window encompassed six major Ebola outbreaks: the 2014–2016 West Africa epidemic (primarily Guinea, Liberia, and Sierra Leone), the 2018 DRC Équateur outbreak, the 2018–2020 DRC North Kivu/Ituri outbreak, the 2022 Uganda Sudan virus outbreak, the 2025 DRC Kasaï outbreak, and the ongoing 2026 DRC/Uganda Bundibugyo virus outbreak. RSV data were plotted chronologically with outbreak periods highlighted to visualise the relationship between epidemic events and public search interest. These six events were selected as all WHO-declared Ebola outbreaks since 2013 with ≥10 confirmed cases and occurrence within the Google Trends retrieval window. Additional minor events during this period (e.g., the 2021 Guinea resurgence, the 2020 DRC Équateur recurrence) were not analysed separately because they generated no detectable RSV signal; their omission reflects the absence of measurable search interest rather than a judgment about epidemiological importance.
- Comparative term analysis. RSV data were simultaneously retrieved for five related search terms (“Ebola”, “Ebola vaccine”, “Ebola symptoms”, “Ebola treatment”, “Ebola cure”) over the same period. Simultaneous retrieval ensures that RSV values are normalised relative to the same baseline, enabling valid cross-term comparisons.
- Misinformation term analysis. To quantify temporal patterns in misinformation-related search behaviour, RSV data were retrieved for four terms associated with Ebola misinformation: “Ebola conspiracy”, “Ebola fake”, “Ebola hoax”, and “Ebola natural cure”. These terms were selected based on their recurrence in published Ebola misinformation taxonomies [16,20] and their presence in the autocomplete data.
- Regional interest analysis. Country-level RSV data for “Ebola” were retrieved for the full study period to identify geographic patterns in search interest. Countries were ranked by aggregate RSV and the top 20 were reported.
- Related queries. Google Trends “related queries” (both “top” and “rising” categories) for the search term “Ebola” were retrieved for the period 1 January 2012 to 29 July 2026 to capture emergent search topics associated with the most recent outbreak cycle.

### 2.6. Statistical Analysis

Descriptive statistics were used to summarise the thematic distribution of autocomplete queries, reported as absolute frequencies and percentages. Cross-tabulations were generated to compare category distributions between English and French queries. For Google Trends data, peak RSV, mean RSV, median RSV, and duration (in weeks) were calculated for each outbreak period. To assess the relationship between overall Ebola search interest and misinformation-related search behaviour, the distribution of all RSV variables was first assessed for normality using the D’Agostino–Pearson K^2^ omnibus test. All variables exhibited severe departures from normality, characterised by extreme positive skewness (range: 9.88–12.65) and leptokurtosis (range: 105.56–158.01), consistent with the spike-and-decay temporal pattern of outbreak-driven search behaviour. On this basis, Spearman’s rank correlation coefficient (ρ) was selected as the primary measure of monotonic association between the monthly RSV for ‘Ebola’ and each misinformation-related search term across the full study period (*n* = 163 monthly observations). Pearson’s correlation coefficient (r) is additionally reported for comparability with prior literature but should be interpreted with caution given the non-normal distributions. To assess potential temporal autocorrelation, the Durbin–Watson (DW) test statistic was computed for the residuals of each bivariate association.

Differences in the proportional distribution of thematic categories between English and French queries were assessed using Pearson’s chi-square test with Yates’ continuity correction applied to individual 2 × 2 comparisons. Cramér’s V was calculated as an effect size measure for the overall association. The conventional threshold of *p* < 0.05 was used as a benchmark for identifying noteworthy distributional differences, with Bonferroni correction applied to the 13 individual category comparisons (adjusted threshold: α = 0.05/13 = 0.0038). Because individual autocomplete suggestions are not statistically independent observations, these *p*-values should be interpreted as descriptive summary measures rather than formal tests of significance. All analyses were conducted in Python 3 using Google Colaboratory. The following Python libraries were used: pandas (data manipulation), matplotlib and seaborn (visualisation), scipy (statistical tests), pytrends (Google Trends API interface), and requests (HTTP queries for autocomplete data).

### 2.7. Methodological Considerations

Several methodological considerations should be noted. First, Google Autocomplete outputs are algorithmically generated and may be influenced by personalisation, temporal trends, and regional factors, although the reproducibility safeguards described above were implemented to mitigate these effects. Second, the keyword-matching classification algorithm, while validated through inter-rater reliability testing, involves a degree of subjectivity in keyword selection and category boundary definition. Third, Google Trends RSV data represent relative, not absolute, search volumes, and are subject to sampling variability, particularly for low-volume queries. Fourth, the study captures search engine behaviour only and does not assess social media activity, offline information-seeking, or the quality of information that users ultimately encounter. Fifth, A methodological caveat applies to the chi-square analyses: individual autocomplete suggestions are not statistically independent observations, as they are nested within seed terms and alphabetical suffixes and are generated by a common algorithm. The chi-square tests should therefore be interpreted as descriptive summaries of the distributional differences within the collected suggestion set, rather than as formal inferential tests generalising to a broader population of potential queries. Finally, the analysis is limited to Google, which, while dominant globally, may underrepresent populations that predominantly use alternative search engines (e.g., Yandex, Baidu). These limitations are discussed further in Section 4.

## 3. Results

### 3.1. Overview of Data Collection

A total of 7347 unique autocomplete suggestions were extracted from 83 seed terms (47 English, 36 French), each expanded with 26 alphabetical suffixes. Of these, 3851 (52.4%) were English-language queries and 3496 (47.6%) were French-language queries. After automated thematic classification, 5403 queries (73.5%) were assigned to one of the 12 predefined categories, while 1944 (26.5%) remained unclassified. Further inspection of the unclassified queries revealed that the majority comprised geographic variants (e.g., “ebola Kenya”, “ebola Nigeria”), proper nouns (e.g., band names, film titles), or queries too ambiguous to categorise reliably (ebola; ebola virus; ebola actualités).

### 3.2. Thematic Distribution of Autocomplete Queries

**Figure 1:**
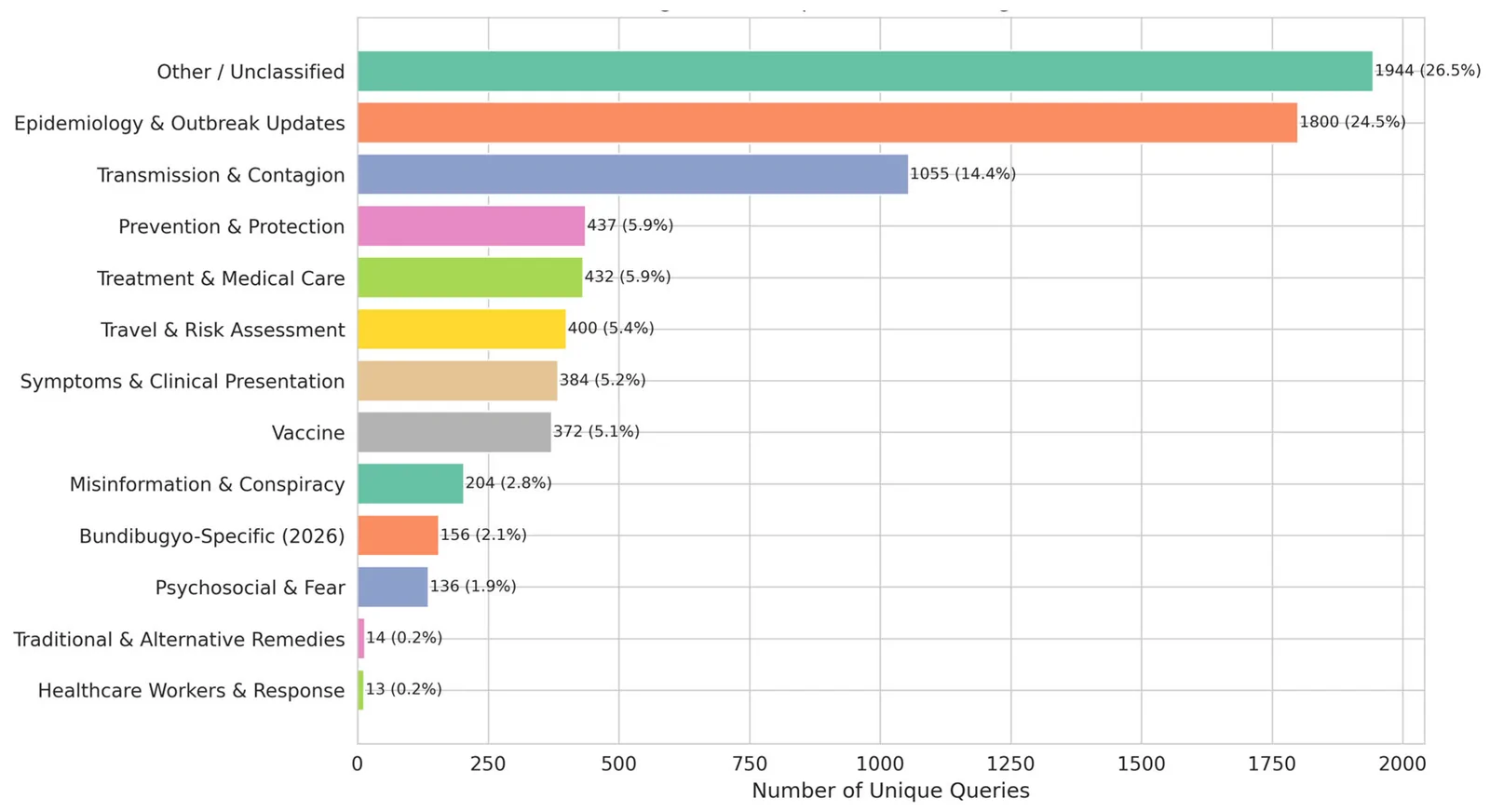
Thematic Distribution of Queries.

The thematic distribution of classified queries is presented in Table 2 and Figure 1. Epidemiology and Outbreak Updates was the dominant category, accounting for 1800 queries (24.5% of total), reflecting widespread public interest in case counts, death tolls, outbreak locations, and official updates. Transmission and Contagion was the second most prevalent category (*n* = 1055; 14.4%), encompassing questions about how Ebola spreads, its routes of transmission, whether it is airborne, and the role of animal reservoirs. Prevention and Protection (*n* = 437; 5.9%) and Treatment and Medical Care (*n* = 432; 5.9%) followed in near-equal proportions, indicating a balanced demand for both protective and therapeutic information.

Travel and Risk Assessment queries (*n* = 400; 5.4%) were notably frequent, reflecting public concern about the geographic spread of Ebola to non-endemic regions, travel safety, and personal risk. Symptoms and Clinical Presentation (*n* = 384; 5.2%) and Vaccine-related queries (*n* = 372; 5.1%) were also well-represented. Misinformation and Conspiracy queries accounted for 204 suggestions (2.8%), including terms related to “Ebola zombie”, conspiracy theories about deliberate creation, and claims of non-existence. Bundibugyo-specific queries (*n* = 156; 2.1%) captured emerging interest in the 2026 outbreak’s novel viral species. Psychosocial and Fear-related queries (*n* = 136; 1.9%) were less frequent than expected, while Traditional and Alternative Remedies (*n* = 14; 0.2%) and Healthcare Workers and Response (*n* = 13; 0.2%) represented the smallest categories.

A secondary descriptive sub-classification of these unclassified queries revealed that the largest sub-group comprised geographic and location variants (*n* = 901; 46.3% of unclassified), such as ‘ebola Kenya’ or ‘ebola afrique’, which represent country- or region-specific versions of epidemiological interest. A further 745 queries (38.3%) were truly ambiguous (e.g., single-word queries like ‘ebola’ or ‘ebola virus’ that lack sufficient specificity for thematic assignment). Smaller sub-groups included media and entertainment references (*n* = 95; 4.9%), definition and education queries (*n* = 76; 3.9%), health system and policy queries (*n* = 51; 2.6%), epidemiological metrics (*n* = 51; 2.6%), and science and biology queries (*n* = 25; 1.3%). The predominance of geographic variants among unclassified queries suggests that this category does not contain a hidden reservoir of misinformation or fear-related content that would alter the primary thematic findings; rather, it reflects the inherently geographic nature of much Ebola-related information-seeking.

### 3.3. Comparison Between English and French Queries

The overall chi-square test indicated a substantial distributional difference between language and query category (χ^2^ = 471.92, df = 12, *p* < 0.001; Cramér’s V = 0.253). Individual category comparisons, with Bonferroni correction for 13 tests (adjusted α = 0.0038), are reported in Table 3. Epidemiology and Outbreak Updates constituted a larger share of English-language suggestions (*n* = 1193; 31.0%) than French (*n* = 607; 17.4%; χ^2^ = 182.9, *p* < 0.001). French-language suggestions showed higher proportions in several categories: Misinformation and Conspiracy accounted for 172 suggestions in French (4.9%) compared to 32 in English (0.8%; χ^2^ = 112.0, *p* < 0.001), Psychosocial and Fear was more prevalent in French (*n* = 121; 3.5%) than in English (*n* = 15; 0.4%; χ^2^ = 93.5, *p* < 0.001), and Prevention and Protection was more heavily represented in French (*n* = 295; 8.4%) than in English (*n* = 142; 3.7%; χ^2^ = 73.1, *p* < 0.001). Bundibugyo-Specific suggestions were proportionally more frequent in French (*n* = 94; 2.7%) than in English (*n* = 62; 1.6%; χ^2^ = 9.8, *p* = 0.002). Travel and Risk Assessment also differed between languages (English: 6.4%; French: 4.4%; χ^2^ = 14.4, *p* < 0.001). Symptoms and Clinical Presentation (English: 5.8%; French: 4.6%; χ^2^ = 5.4, *p* = 0.020) and Traditional and Alternative Remedies (English: 0.1%; French: 0.3%; χ^2^ = 6.7, *p* = 0.010) showed nominal differences that did not meet the Bonferroni-adjusted threshold. The proportions of Vaccine-related (English: 4.8%; French: 5.3%; χ^2^ = 1.0, *p* = 0.312), Transmission and Contagion (English: 14.9%; French: 13.7%; χ^2^ = 2.1, *p* = 0.152), Treatment and Medical Care (English: 6.0%; French: 5.7%; χ^2^ = 0.4, *p* = 0.547), and Healthcare Workers and Response (English: 0.3%; French: 0.1%; χ^2^ = 2.2, *p* = 0.135) suggestions did not show substantial differences between language sets. A higher proportion of French-language suggestions remained unclassified (29.0% vs. 24.2%; χ^2^ = 21.5, *p* < 0.001).

### 3.4. Temporal Dynamics of Ebola-Related Search Interest

**Figure 2:**
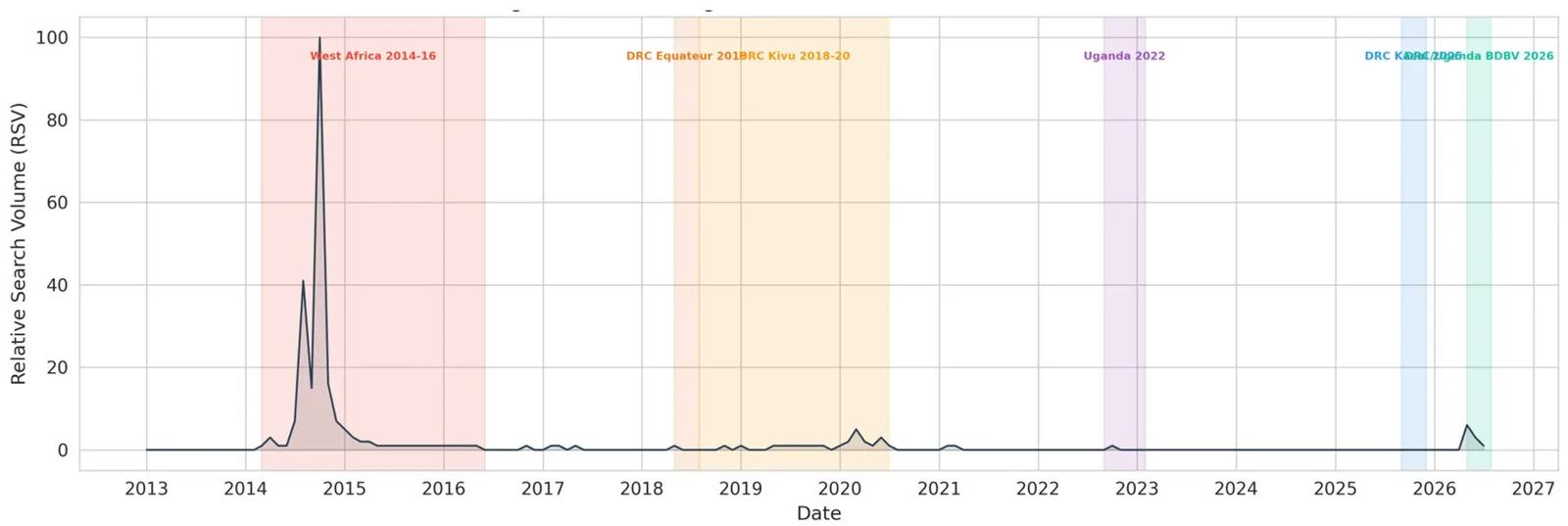
Comparative search terms over time.

Google Trends data spanning January 2013 to July 2026 revealed highly episodic patterns of global search interest for “Ebola” (Figure 2). The 2014 West Africa epidemic generated by far the largest search spike, with a peak RSV of 100 in October 2014 (Table 4). However, despite its scale and severity, the mean RSV across the 2014–2016 outbreak period was only 7.8, reflecting a sharp initial peak followed by rapid decline, a pattern of diminishing search interest that has been observed in prior infodemiological studies of outbreak-related search activity. The 2018–2020 DRC North Kivu/Ituri outbreak, the country’s second-largest, registered a comparatively modest peak RSV of 5, with a mean of 1.0 across 23 weeks, suggesting substantially lower global attention despite a prolonged epidemic. The 2022 Uganda and 2025 DRC Kasaï outbreaks generated minimal global search activity (peak RSV ≤ 1).

The ongoing 2026 Bundibugyo virus outbreak showed a peak RSV of 6, with a mean of 3.3 over its initial three weeks of data; notably higher sustained interest than the much larger 2018–2020 Kivu outbreak. This may reflect the novelty of the Bundibugyo species, the PHEIC declaration, the absence of a licensed vaccine, cross-border spread to Uganda or closer attention to epidemics in the post-COVID-19 era.

### 3.5. Comparative Search Term Analysis

When the RSV for “Ebola” was compared with related search terms (“Ebola vaccine”, “Ebola symptoms”, “Ebola treatment”, “Ebola cure”), all terms exhibited similar temporal patterns, characterised by sharp spikes during outbreak events followed by rapid return to baseline. During the 2014 peak, “Ebola symptoms” and “Ebola cure” showed the highest relative interest alongside the core term, suggesting that the public’s dominant information needs during the initial phase of an outbreak centre on recognising the disease and identifying potential treatments. “Ebola vaccine” interest, while present, was proportionally lower during 2014, likely reflecting the limited availability of candidate vaccines at that time, but showed increased relative prominence during the 2018–2020 Kivu outbreak, coinciding with the deployment of the rVSV-ZEBOV (Ervebo) vaccine in a ring vaccination strategy.

### 3.6. Misinformation-Related Search Trends

Prior to correlation analysis, the D’Agostino–Pearson K^2^ normality test confirmed that all RSV variables were severely non-normally distributed (Table 5). The ‘Ebola’ RSV showed a skewness of 9.88 and kurtosis of 105.56 (K^2^ = 313.32, *p* < 0.001), reflecting the extreme spike-and-decay temporal profile driven by the October 2014 search peak. All comparative and misinformation-related terms showed similarly extreme departures (all K^2^ > 300, all *p* < 0.001). Spearman’s rank correlation was therefore adopted as the primary association measure.

Temporal analysis of misinformation-associated search terms (“Ebola conspiracy”, “Ebola fake”, “Ebola hoax”, “Ebola natural cure”) revealed that these terms closely tracked the overall “Ebola” RSV, with spikes occurring during outbreak events and negligible interest between outbreaks. Correlation analysis indicated strong positive associations between overall Ebola search interest and each misinformation term (Table 6). Spearman rank correlations indicated moderate to strong positive monotonic co-movement between overall Ebola search interest and each misinformation term (*ρ* = 0.359–0.697; all *p* < 0.001), indicating that misinformation-related searches co-vary with overall disease interest across the full range of search activity, not merely during extreme peaks (Table 6). Pearson correlations, reported for comparability with prior literature, were uniformly high (r = 0.937–0.986; all *p* < 0.001) but are influenced by the shared extreme values during the 2014 outbreak peak. ‘Ebola conspiracy’ showed the strongest rank correlation (*ρ* = 0.697), while ‘Ebola natural cure’ showed the weakest (*ρ* = 0.359), possibly reflecting different temporal dynamics: conspiracy queries may persist at low levels between outbreaks, while remedy searches are more exclusively outbreak-driven.

The Durbin–Watson test statistic for the Ebola RSV series was 1.36, indicating moderate positive autocorrelation. This is expected for temporal search data, where weeks of elevated search interest cluster during outbreak peaks. Positive autocorrelation implies that the effective number of independent observations is smaller than the nominal *n* = 163, and the reported *p*-values should therefore be interpreted as approximate rather than exact. Autocorrelation does not bias the magnitude or direction of the correlation coefficients themselves; however, the reported *p*-values underestimate the true uncertainty and should not be interpreted as formal significance tests. The correlations are reported as descriptive measures of observed temporal co-movement within this dataset.

**Table 5:** D’Agostino–Pearson K^2^ normality test results for Google Trends RSV variables.

Variable	K^2^	*p*-Value	Skewness	Kurtosis
Ebola	313.32	9.17 × 10^−69^	9.88	105.56
Ebola vaccine	356.71	3.47 × 10^−78^	12.65	158.01
Ebola symptoms	330.76	1.50 × 10^−72^	10.91	126.02
Ebola treatment	—	—	—	— (constant = 0)
Ebola cure	356.71	3.47 × 10^−78^	12.65	158.01

### 3.7. Geographic Distribution of Search Interest

Country-level analysis of cumulative RSV for “Ebola” across the 2013–2026 period identified the three countries most directly affected by the 2014–2016 epidemic as the highest-ranking: Liberia (RSV = 100), Sierra Leone (RSV = 90), and Guinea (RSV = 37). Among the top 20 countries, several West and Central African nations with direct or indirect outbreak exposure were represented, including Mali (RSV = 13), Uganda (RSV = 12), and Equatorial Guinea (RSV = 16). The Gambia (RSV = 13), Eritrea (RSV = 11), and Anguilla (RSV = 11) also ranked highly.

### 3.8. Outbreak-Specific Search Interest Profiles

When search interest curves were aligned by outbreak start date, distinct patterns emerged across the six outbreak periods. The 2014–2016 West Africa epidemic showed a dramatic spike-and-decay profile, with interest peaking approximately seven months after outbreak onset and declining to near-baseline within 12 months despite ongoing transmission. The 2018–2020 DRC Kivu outbreak displayed a lower-amplitude but more sustained pattern, with a secondary peak in early 2020, likely coinciding with the declaration of the COVID-19 pandemic, which generated renewed interest in all infectious disease topics. The 2026 Bundibugyo outbreak, though captured over only three weeks at the time of data collection, showed the steepest initial ascent of any post-2014 outbreak, consistent with the PHEIC declaration and international media attention.

### 3.9. Notable Query Patterns

Several noteworthy patterns emerged from the autocomplete data that warrant individual mention. First, the “Ebola zombie” cluster (appearing in both English and French) represented the single most distinctive misinformation theme, with multiple variants suggesting sustained public interest in a fictitious association between Ebola and zombie-like reanimation. This term, and its classification as misinformation, warrants further thought. While these queries contain factually incorrect premises about Ebola’s clinical effects, the zombie trope occupies a complex position at the intersection of misinformation, cultural expression, and entertainment media. The association between haemorrhagic fever and zombie imagery has roots in both Haitian vodou traditions, where the concept of the zombi carries specific cultural and spiritual significance distinct from its Western horror-genre usage, and in popular entertainment. A proportion of these queries may therefore reflect entertainment-driven curiosity rather than genuine belief in Ebola-induced reanimation. However, from an infodemic management perspective, the distinction is functionally less important than it might appear: regardless of the searcher’s intent, these queries are surfaced by Google Autocomplete to all users entering Ebola-related terms, potentially reinforcing false associations for populations with limited health literacy. We therefore retained the misinformation classification while acknowledging its heterogeneous origins.

Second, Bundibugyo-specific queries such as “ebola new strain”, “ebola different type”, and “no vaccine bundibugyo” reflected growing public awareness of the taxonomic diversity of ebolaviruses and the absence of a licensed vaccine for the 2026 outbreak. Third, a substantial cluster of travel-related queries referencing countries far from the outbreak zone (e.g., “ebola Australia”, “ebola China”, “ebola Canada”, “ebola India”) revealed a pattern of risk assessment behaviour among populations with negligible objective risk; a hallmark of infodemic-driven anxiety. Fourth, the near-absence of healthcare worker queries (*n* = 13; 0.2%) and psychosocial support queries (*n* = 136; 1.9%) suggests that the public’s online information-seeking is overwhelmingly oriented toward biomedical and epidemiological concerns, with limited spontaneous demand for content addressing the human dimensions of the epidemic response.

## 4. Discussion

This study set out to understand what the world asks when it hears the word “Ebola”. By combining the granularity of Google Autocomplete, which captures the actual phrases people type into a search bar, with the temporal depth of Google Trends, we were able to map both the content and the timing of public information demand across six Ebola outbreaks spanning more than a decade. The resulting dataset of 7347 unique queries in two languages, together with 163 months of trend data, offers what is, to our knowledge, the most comprehensive infodemiological portrait of the public information landscape around Ebola virus disease to date.

### 4.1. The Suggestion Landscape Reflects Risk Quantification over Practical Guidance

The dominance of Epidemiology and Outbreak Updates (24.5%) and Transmission and Contagion (14.4%) in the autocomplete data tells a consistent story: during Ebola outbreaks, the suggestion landscape is dominated by risk quantification. Queries like “ebola death rate”, “ebola how does it spread”, “ebola airborne”; these are the questions of a population performing its own risk assessment, often in the absence of clear, accessible official communication [34]. This pattern mirrors what Alicino et al. [28] observed during the 2014 West Africa epidemic, when global search volumes for “Ebola” surged in proportion to media coverage rather than to epidemiological reality. What our data add is the qualitative dimension: it is not merely that people search more during outbreaks, but that they search for specific reassurance about transmission routes, mortality figures, and geographic proximity, revealing an information gap in the basics of the disease that may reflect a lack of adequate and informative media context, public education strategies or overall health literacy.

The prominence of Travel and Risk Assessment queries (5.4%) reveals another facet of the need for reassurance in the international context. A substantial cluster of queries referenced countries far from any outbreak zone, “ebola Australia”, “ebola Canada”, “ebola China”, “ebola India”, revealing that populations with negligible objective risk nevertheless engage in active information-seeking driven by perceived threat. Towers et al. [29] conceptualised this phenomenon as a “contagion of fear” during the 2014 US Ebola events, where search behaviour followed epidemic dynamics with its own reproduction number and recovery rate. Our data suggest that this pattern recurs with each new outbreak, albeit in different proportions, regardless of the actual epidemiological risk to distant populations, and may represent a stable feature of the global response to high-fatality infectious diseases.

Strikingly, the categories one might expect to be most useful to populations actually affected by Ebola, Healthcare Workers and Response (0.2%) and Psychosocial and Fear (1.9%), were among the least represented. This gap is not merely an artefact of keyword selection; it reflects a genuine asymmetry between what health authorities prioritise in outbreak communication (safe burial, psychosocial support, healthcare worker protection) and what the public spontaneously seeks online (case counts, transmission mechanisms, personal risk). The International Federation of Red Cross and Red Crescent Societies’ community feedback report from the 2026 DRC outbreak identified a similar disconnect, noting that affected communities’ primary concerns centred on “fear, misinformation and mistrust” rather than the practical guidance that response agencies were delivering [35]. This finding supports previous studies that showed that the messages international and national agencies try to convey were not being reached or trusted by the public by showcasing that people are not showing interest in services provided; this has direct implications for the design of health communication strategies, which should be structured around trusted community members and wide reaching channels on the questions people are actually asking rather than the messages responders wish to convey [36].

### 4.2. Language-Based Differences in the Suggestion Landscape

The most notable descriptive finding within the autocomplete data is the divergence between the English-language and French-language suggestion sets. French-language searches contained six times more misinformation and conspiracy content (4.9% vs. 0.8%) and nearly nine times more psychosocial and fear-related queries (3.5% vs. 0.4%). Traditional and alternative remedy queries, while small in absolute numbers, were almost exclusively French. These differences are unlikely to reflect random variation in keyword matching; they point to fundamentally different information ecosystems surrounding Ebola in Anglophone and Francophone contexts.

However, we acknowledge that the observed language-based differences cannot be attributed to language per se, independently of the geographic, cultural, and socioeconomic contexts in which these languages are used. In the context of Ebola, English and French function as proxies for fundamentally different populations: French is the primary language of the DRC, which has experienced 17 outbreaks since 1976 and is the epicentre of the 2026 Bundibugyo outbreak, while English-language queries are more likely to originate from geographically distant populations with indirect exposure. Furthermore, the populations captured through internet search data in the DRC represent a multiply selected subset of the overall population: individuals with sufficient literacy and digital literacy, reliable internet access, a suitable device, and in the case of English-language searches, proficiency in a second language. The observed Francophone–Anglophone differences may therefore partly reflect differences in the sociodemographic characteristics of the populations generating these searches, with English-language DRC searchers potentially representing a more educated, internationally connected subgroup, rather than differences exclusively attributable to language-specific information environments. These overlapping forms of selection bias should be considered when interpreting the language-based comparisons and underscore the need for complementary offline methods (community surveys, radio content analysis, community health worker feedback) to validate the patterns observed in online search data.

This pattern is best understood against the backdrop of the communities most directly affected by recurrent Ebola outbreaks; however, it must be noted that these ideas draw on contextual literature from epidemiological studies, surveys, and field reports, not just the findings produced by the autocomplete analysis itself. The DRC, which has experienced 17 outbreaks since 1976 [6], is a Francophone country where decades of armed conflict, exploitation of mineral wealth by external actors, and the near-total collapse of state services across rural Ituri and North Kivu have eroded institutional trust to a degree that is difficult to overstate [18]. A population-based survey conducted during the 2018–2019 North Kivu outbreak found that fewer than one in three residents trusted local authorities, and those who harboured mistrust were significantly less likely to accept Ebola vaccination or seek formal healthcare [18]. When the same communities witnessed two Ebola treatment centres set ablaze within days of the 2026 outbreak declaration [37], the fire was not fuelled only by ignorance but by accumulated grievance against the institutions claiming to address it. As The New Humanitarian argued in its analysis of the 2026 response, “this is a crisis of history, not misinformation” [38]; a framing that the search data appear to corroborate, as French-language users disproportionately searched for content questioning the legitimacy of the response rather than seeking basic biomedical information.

If the observed difference between language-specific suggestion sets reflects, even partially, differences in the information environments encountered by users in these languages, the practical implications are considerable: infodemic management strategies designed in Anglophone contexts, where misinformation-related suggestions constitute less than 1% of the autocomplete landscape, may be inadequate for French-language populations where such content is substantially more prevalent. Health authorities operating in the DRC and neighbouring Francophone countries should invest in French-language digital health literacy programmes, partner with trusted community health workers who can mediate between online information and lived experience [36,39], and critically, address the structural determinants of mistrust rather than treating misinformation as a purely communicative problem.

### 4.3. Declining Search Interest Across Successive Outbreaks

The Google Trends data revealed a phenomenon with troubling implications for global health preparedness: each successive Ebola outbreak was associated with lower peak RSV values than the last, though this pattern may reflect multiple factors including declining media novelty, differences in outbreak severity, changes in Internet penetration, and competing global health events rather than a single mechanism of attention decay. The 2014 West Africa epidemic, the largest Ebola outbreak in history, produced a peak RSV of 100 and dominated global search behaviour for months. Yet the 2018–2020 DRC Kivu outbreak, which killed 2299 people and persisted for nearly two years [7], generated a peak RSV of only 5. The 2022 Uganda outbreak (164 cases) and the 2025 DRC Kasaï outbreak barely registered at all. This pattern of diminishing returns is consistent with the concept of “compassion fatigue” or “crisis normalisation” described in disaster communication research [40], where repeated exposure to a threat reduces its perceived novelty and, consequently, the public’s information-seeking response.

The 2026 Bundibugyo outbreak partially breaks this pattern, with a peak RSV of 6 and a mean of 3.3 over its initial weeks, higher sustained interest than the much larger Kivu outbreak. Several factors may explain this relative resurgence: the novelty of the Bundibugyo species, which the public appears to have recognised as distinct (“ebola new strain”, “ebola different type”); the PHEIC declaration, which was widely covered in international media; the absence of a licensed vaccine, which introduced a new dimension of uncertainty [10,13]; the cross-border spread to Uganda, which expanded the geographic footprint of concern and possible changes to infectious disease perception and anxiety in the post-COVID-19 era. These findings suggest that information-seeking behaviour is not driven solely by case counts or mortality but by perceived novelty and uncertainty; a lesson that health communicators should internalise when planning outbreak messaging.

The secondary peak observed in Ebola search interest during early 2020 warrants separate comment. This spike coincides not with any Ebola event but with the declaration of the COVID-19 pandemic, which appears to have generated a “spillover” of public interest in other epidemic-prone diseases. This observation is consistent with findings by Mheidly and Fares [41], who demonstrated that COVID-19 awareness increased public interest in multiple infectious diseases simultaneously. For health communicators, this represents an opportunity: periods of heightened general awareness of infectious disease may be optimal windows for reinforcing Ebola preparedness messages, even in the absence of an active outbreak.

We acknowledge that the declining RSV trend cannot be attributed to any single mechanism. The observed pattern is consistent with what has been, in the previous literature, described as attention decay, but equally consistent with alternative explanations including reduced media coverage of subsequent outbreaks, geographic remoteness of later outbreaks relative to international media centres, improvements in outbreak response that reduce the duration of public alarm, or changes in the competitive information environment (e.g., the COVID-19 pandemic dominating the public health news cycle from 2020 onward). Disentangling these factors would require integration of media content analysis, Internet penetration data, and epidemiological covariates, which was beyond the scope of this descriptive study.

### 4.4. Misinformation as a Constant Companion

The strong positive co-movement between overall Ebola search interest and misinformation-related queries carries an uncomfortable but important message: misinformation does not arise independently of legitimate information-seeking; it is embedded within it. When people search for “Ebola”, a predictable proportion simultaneously search for “Ebola conspiracy”, “Ebola hoax”, and “Ebola natural cure”. This is not a parallel phenomenon that can be addressed separately; it is an integral component of the public’s response to epidemic threats [14,20].

The differential Spearman correlations are instructive. “Ebola conspiracy” showed the strongest rank correlation (*ρ* = 0.697), indicating sustained interest across varying levels of search activity. “Ebola natural cure”, by contrast, showed the weakest (*ρ* = 0.359), suggesting that natural remedy searches are more spike-dependent, arising primarily during peak outbreak attention and receding more rapidly than conspiracy queries. This distinction has practical relevance: conspiracy narratives may require sustained counter-messaging, while remedy-related misinformation may be more effectively addressed through targeted interventions during the acute phase of an outbreak.

### 4.5. The Bundibugyo Factor: When Novelty Reshapes the Infodemic

The emergence of a Bundibugyo-specific query cluster (2.1% of all queries) within weeks of the 2026 outbreak declaration is, to our knowledge, the first documentation of real-time public awareness of ebolavirus species diversity. Queries such as “ebola new strain”, “ebola different type”, “no vaccine bundibugyo”, and “ebola virus species” indicate that the public is not treating Ebola as a monolithic entity but is beginning to differentiate between viral species; a level of granularity that was entirely absent from the search landscape during previous outbreaks. This represents both an opportunity and a risk: an opportunity because informed publics are better positioned to understand why prior vaccines may not provide protection; a risk because the “new strain” framing can amplify fear and fuel conspiracy narratives about engineered pathogens [21,42].

Health authorities should respond to this emerging awareness proactively by providing clear, accessible communication about the taxonomy of ebolaviruses, the specific characteristics of Bundibugyo virus, and the status of countermeasure development, rather than allowing the information vacuum to be filled by speculative or misleading content [13].

### 4.6. Comparison with Prior Infodemiological Studies

Our findings both extend and challenge the conclusions of Alicino et al. [28], who reported a strong correlation between Ebola-related Google search volumes and weekly case counts during the 2014 epidemic. While our temporal data confirm the existence of outbreak-driven search spikes, the multi-outbreak comparison reveals that the case-count/search-volume relationship is far from linear: the 2018–2020 Kivu outbreak, with 3481 cases, generated a fraction of the search interest produced by the 2014 West Africa epidemic. This suggests that global search behaviour is shaped more by media amplification and perceived novelty than by epidemiological burden; a conclusion anticipated by Towers et al. [29] in their “contagion of fear” model but not previously tested across multiple Ebola events.

Our study also extends the social media analyses of Oyeyemi et al. [17] and Sell et al. [16], who identified misinformation themes on Twitter during the 2014 epidemic. While those studies characterised the supply side of misinformation (what is posted), our autocomplete data capture the demand side (what is sought). The two perspectives are complementary: if Twitter studies show that “Ebola zombie” content circulates widely, our data confirm that users are actively searching for it. The convergence of supply and demand around specific misinformation themes, particularly the “zombie” narrative and conspiracy theories about deliberate creation, suggests that these themes resonate with deep-seated cultural anxieties that transcend specific outbreaks or media platforms.

### 4.7. Limitations

Several limitations should be considered when interpreting these findings. First, Google Autocomplete suggestions are algorithmically generated and subject to temporal, geographic, and personalisation effects. Although reproducibility safeguards were implemented, the dynamic nature of the algorithm means that exact replication at a later date would yield partially different results. Second, the keyword-matching classification algorithm, while validated through inter-rater reliability testing, is inherently limited by the keywords selected; queries containing relevant content in unexpected phrasings may have been misclassified or left unclassified. The relatively high proportion of unclassified queries (26.5%), many of which were geographic variants, suggests that future studies may benefit from a supplementary machine learning classification step. Third, Google Trends data represent relative rather than absolute search volumes and are normalised within the queried time frame, which limits direct comparisons of absolute interest across periods. Fourth, the study captures only Google-mediated search behaviour, which may underrepresent populations that rely on alternative search engines, social media platforms, or offline information channels; a particularly relevant limitation for the DRC, where internet penetration remains limited and community health workers and radio serve as primary information sources [39]. Fifth, Google Trends data for French-language terms could not be fully retrieved due to API rate limitations, and our temporal analyses therefore reflect predominantly English-language search behaviour. Sixth, the absence of French-language Google Trends data represents a significant limitation. The temporal analyses presented here reflect predominantly English-language search behaviour, and we cannot confirm that the temporal patterns, including the misinformation co-movement, attention decay, and outbreak-specific profiles, generalise to Francophone populations. French-language search terms for narrow misinformation concepts consistently returned insufficient data volumes for meaningful temporal analysis via the Google Trends API. The populations most directly affected by the 2026 Bundibugyo outbreak are predominantly Francophone, making this gap particularly noteworthy. Furthermore, we did not perform regression modelling of the relationship between daily case counts and search volumes, due to the mismatch between the temporal resolution of available data sources (monthly RSV vs. irregularly reported case counts), the substantial autocorrelation in both series (DW = 1.36 for Ebola RSV), and the incompleteness of case count data for the ongoing Bundibugyo outbreak. Time-series regression incorporating lagged search volumes as potential leading indicators of case reports represents a valuable direction for future research once finalised epidemiological data become available. Additionally, the correlation between outbreak events and search interest is observational and does not establish causation; media coverage, policy announcements, and social media virality are likely mediating variables that were not measured. Finally, the study analyses what people search for, not whether they find accurate information or how search behaviour translates into health-related decisions.

### 4.8. Implications for Practice and Policy

Despite these limitations, the findings carry several actionable implications. First, health communication strategies should be designed around the questions people are actually asking, particularly those related to transmission routes, personal risk, and outbreak trajectory, rather than around the messages that health authorities wish to deliver. Second, the pronounced language divide in misinformation content demands targeted French-language infodemic management interventions in Francophone African countries, with recognition that misinformation in these contexts is rooted in structural mistrust rather than informational deficit [38]. Third, the temporal comovement between overall search interest and misinformation queries means that counter-misinformation efforts should be deployed at the earliest possible stage of an outbreak, before conspiracy narratives become entrenched. Fourth, the emergence of Bundibugyo-specific queries demonstrates that the public can and does differentiate between viral species, and health communicators should embrace this granularity rather than defaulting to generic “Ebola” messaging. Fifth, the attention decay pattern across successive outbreaks highlights the need for sustained, inter-epidemic communication strategies that maintain baseline public awareness of Ebola, particularly in endemic regions.

### 4.9. Lessons for the Ongoing Bundibugyo Virus Disease Response

The patterns observed in this study, considered alongside evidence from prior research, carry implications for the ongoing 2026 Bundibugyo virus disease outbreak that warrant separate consideration. First, the six-fold excess of misinformation and conspiracy content in French-language suggestions (4.9% vs. 0.8%), while not directly attributable to specific populations, is consistent with a more misinformation-laden information environment for French-language searchers during this outbreak. While this pattern describes differences between suggestion sets rather than between defined populations, it points to a language-specific dimension of information demand that warrants attention in outbreak communication planning. Counter-messaging efforts for the Bundibugyo response should therefore prioritise French-language content creation and dissemination from the outset, rather than translating Anglophone materials after the fact, as has been the default in prior responses [36].

Second, the emergence of a distinct Bundibugyo-specific query cluster (“ebola new strain”, “no vaccine bundibugyo”, “ebola different type”) reveals that the public is actively aware that this outbreak involves a viral species for which the established countermeasures, most notably the Ervebo (rVSV-ZEBOV) vaccine, do not apply [13]. This awareness creates both an opportunity and a vulnerability. If health authorities do not proactively communicate the current status of Bundibugyo virus countermeasure development, including the pipeline status of candidate vaccines and monoclonal antibody therapies [13,43], the resulting information vacuum will predictably be filled by conspiracy narratives linking the absence of a vaccine to deliberate neglect or population-level experimentation; narratives already documented in the autocomplete data and in field reports from the current response [21,38].

Third, the strong temporal co-movement between misinformation-related queries and overall search interest (Spearman ρ = 0.359–0.697) suggests that as the Bundibugyo outbreak continues to attract global attention, misinformation-related search activity is likely to increase in parallel. The window for establishing authoritative, accessible, and linguistically appropriate information channels is therefore the early phase of the outbreak, before search interest peaks and the misinformation-to-information ratio becomes entrenched. WHO’s infodemic management programme has articulated a similar principle of early intervention [44], but our data provide the first quantitative evidence that this window is real and measurable for Ebola outbreaks specifically.

Operationally, these findings suggest several concrete steps for communication strategies during the ongoing Bundibugyo response. First, French-language content should be developed as primary material rather than translated from English-language originals, as the thematic priorities of Francophone searchers differ from those of Anglophone populations. Second, content addressing the Bundibugyo virus species specifically, including its relationship to other ebolaviruses, the absence of a licensed vaccine, and the pipeline status of candidate countermeasures [13,43], should be prioritised, given the high proportion of Bundibugyo-specific queries in French. Third, community health workers and trusted local media, particularly radio, should be engaged as intermediaries between online information and community understanding, following the social mobilisation model documented during the West Africa response [36]. Fourth, the strong temporal coupling between search interest and misinformation queries indicates that counter-messaging efforts should be deployed at the earliest phase of any outbreak, before conspiracy narratives become entrenched in the autocomplete ecosystem. Finally, the structural determinants of mistrust in the DRC, including the legacy of armed conflict, external resource extraction, and prior response failures [18,38], must be addressed as a prerequisite for effective communication, rather than treating information gaps as purely communicative problems.

Finally, the near-absence of healthcare worker and psychosocial support queries (together comprising only 0.4% of total queries) in the context of an outbreak that has already seen targeted violence against response personnel [19] suggests a dangerous disconnect between the severity of the crisis facing frontline workers and the public’s awareness of or interest in their situation. Communication strategies for the Bundibugyo response should actively surface healthcare worker narratives and psychosocial support resources, rather than waiting for spontaneous public demand that the data suggest will not materialise.

Finally, the progressive attention decay observed across six outbreaks, with each successive event generating less global search interest than the last, poses a specific risk for the Bundibugyo response: despite being the first PHEIC-designated Ebola event caused by a species lacking a licensed vaccine, the 2026 outbreak has generated a peak RSV of only 6 compared to 100 during the 2014 West Africa epidemic. This erosion of global attention has direct consequences for resource mobilisation, political commitment, and the sustained international engagement that prolonged outbreak responses require. Advocacy strategies should explicitly leverage the novelty of the Bundibugyo species and the vaccine gap, the very factors driving the Bundibugyo-specific query cluster, to counteract the normalisation of Ebola as a recurring but low-priority crisis.

## 5. Conclusions

This study demonstrates that Google Autocomplete and Google Trends, used in combination, can provide a detailed, real-time portrait of the public information landscape during infectious disease outbreaks. Applied to Ebola, these tools reveal a landscape dominated by risk quantification and transmission concerns, accompanied by misinformation-related content that co-moves with overall search interest, and characterised by substantial differences between English-language and French-language suggestion sets. The attention decay observed across successive outbreaks, coupled with the partial resurgence of interest during the novel 2026 Bundibugyo event, suggests that perceived novelty and uncertainty, rather than epidemiological severity, are the primary drivers of global information-seeking. These findings underscore the need for infodemic management strategies that are not only evidence-based and timely but also linguistically and culturally adapted, structurally informed, and sustained beyond the acute phase of any single outbreak. For the ongoing Bundibugyo virus disease response specifically, our findings suggest that French-language counter-messaging, proactive communication about the countermeasure gap, and early deployment of authoritative information channels are not optional enhancements but essential components of an effective public health response to this unprecedented outbreak.

## Figures and Tables

**Table 1:** Thematic Categories and Descriptions.

Category	Description
Misinformation & Conspiracy	Queries reflecting false claims, conspiracy theories, or deliberate disinformation about Ebola origins, purpose, or existence
Traditional & Alternative Remedies	Queries about non-evidence-based treatments, herbal remedies, spiritual healing, or faith-based interventions
Transmission & Contagion	Queries about how Ebola spreads, routes of transmission, contagiousness, and incubation period
Symptoms & Clinical Presentation	Queries about signs, symptoms, disease stages, and clinical manifestations
Treatment & Medical Care	Queries about evidence-based treatments, therapeutics, hospitalisation, survival, and recovery
Vaccine	Queries about Ebola vaccines, vaccination campaigns, efficacy, side effects, and availability
Prevention & Protection	Queries about personal and community-level protective measures, hygiene, PPE, isolation, and safe burial
Epidemiology & Outbreak Updates	Queries about case counts, death tolls, outbreak locations, timelines, and official updates
Travel & Risk Assessment	Queries about travel safety, travel bans, personal risk, and geographic spread to non-endemic regions
Psychosocial & Fear	Queries reflecting emotional responses, fear, anxiety, stigma, mental health, and community impact
Healthcare Workers & Response	Queries about healthcare worker risk, humanitarian response, and organisational involvement
Bundibugyo-Specific (2026)	Queries specifically referencing the Bundibugyo virus species, the 2026 outbreak, or associated geographic locations

**Table 2:** Thematic distribution of Ebola-related Google Autocomplete queries (*N* = 7347).

Category	*n*	%
Epidemiology & Outbreak Updates	1800	24.5
Transmission & Contagion	1055	14.4
Prevention & Protection	437	5.9
Treatment & Medical Care	432	5.9
Travel & Risk Assessment	400	5.4
Symptoms & Clinical Presentation	384	5.2
Vaccine	372	5.1
Misinformation & Conspiracy	204	2.8
Bundibugyo-Specific (2026)	156	2.1
Psychosocial & Fear	136	1.9
Traditional & Alternative Remedies	14	0.2
Healthcare Workers & Response	13	0.2
Other/Unclassified	1944	26.5
*Geographic/Location*	901	12.3
*Truly Ambiguous*	745	10.1
*Media/Entertainment*	95	1.3
*Definition/Education*	76	1
*Health System/Policy*	51	0.6
*Epidemiological Metrics*	51	0.6
*Science/Biology*	25	0.3

**Table 3:** Thematic distribution of queries by language.

Category	English (*n*)	English (%)	French (*n*)	French (%)	χ^2^	*p* Value	Bonferroni Threshold
Epidemiology & Outbreak Updates	1193	31.0	607	17.4	182.9	<0.001	Sig.
Transmission & Contagion	575	14.9	480	13.7	2.1	0.152	NS
Prevention & Protection	142	3.7	295	8.4	73.1	<0.001	Sig.
Treatment & Medical Care	233	6.0	199	5.7	0.4	0.547	NS
Travel & Risk Assessment	247	6.4	153	4.4	14.4	<0.001	Sig.
Symptoms & Clinical Presentation	224	5.8	160	4.6	5.4	0.020	NS *
Vaccine	185	4.8	187	5.3	1.0	0.312	NS
Misinformation & Conspiracy	32	0.8	172	4.9	112.0	<0.001	Sig.
Bundibugyo-Specific (2026)	62	1.6	94	2.7	9.8	0.002	Sig.
Psychosocial & Fear	15	0.4	121	3.5	93.5	<0.001	Sig.
Trad. & Alternative Remedies	2	0.1	12	0.3	6.7	0.010	NS *
Healthcare Workers & Response	10	0.3	3	0.1	2.2	0.135	NS
Other/Unclassified	931	24.2	1013	29.0	21.5	<0.001	Sig.

Sig. = significant after Bonferroni correction (adjusted α = 0.05/13 = 0.0038). NS = not significant. * Nominally significant (*p* < 0.05) but not significant after Bonferroni correction. Overall χ^2^ = 471.92, df = 12, *p* < 0.001; Cramér’s V = 0.253. Chi-square tests with Yates’ continuity correction. Note: individual autocomplete suggestions are nested within seed terms and generated by a common algorithm; chi-square statistics should be interpreted as descriptive summaries of distributional differences within the suggestion set rather than formal inferential tests.

**Table 4:** Google Trends search interest metrics by outbreak period.

Outbreak	Period	Peak RSV	Mean RSV	Median RSV	Duration (wk)	Peak Date
West Africa 2014–16	Mar 2014–Jun 2016	100	7.8	1.0	28	Oct 2014
DRC Équateur 2018	May–Jul 2018	1	0.3	0.0	3	May 2018
DRC Kivu 2018–20	Aug 2018–Jun 2020	5	1.0	1.0	23	Mar 2020
Uganda 2022	Sep 2022–Jan 2023	1	0.2	0.0	5	Oct 2022
DRC Kasaï 2025	Sep–Dec 2025	0	0.0	0.0	4	—
DRC/Uganda BDBV 2026	May–Jul 2026	6	3.3	3.0	3	May 2026

**Table 6:** Correlation between overall “Ebola” RSV and misinformation-related search terms (*n* = 163 monthly data points).

Search Term	Spearman ρ	*p*-Value	Pearson r	*p*-Value
Ebola conspiracy	0.697	4.62 × 10^−25^	0.966	4.49 × 10^−96^
Ebola fake	0.625	4.66 × 10^−19^	0.959	2.53 × 10^−90^
Ebola hoax	0.496	1.60 × 10^−11^	0.937	2.46 × 10^−75^
Ebola natural cure	0.359	2.47 × 10^−6^	0.986	3.80 × 10^−126^

Nominal *p*-values are reported for transparency. Due to substantial positive temporal autocorrelation (Durbin-Watson = 1.36), the effective degrees of freedom are smaller than n − 2 and the reported *p*-values underestimate the true uncertainty. These correlations describe observed temporal co-movement within this dataset and should not be interpreted as formal tests of significance.

## Data Availability

Google Autocomplete suggestions and Google Trends data are publicly accessible, and the dataset analysed during this study was acquired from these services through a structured script that was run on Google Colab. The complete pipeline is available from the authors upon request.

## Supplementary Materials

The following supporting information can be downloaded at https://www.mdpi.com/article/10.3390/v18090987/s1. Table S1: Complete list of seed terms used for Google Autocomplete query extraction; Table S2: Classification keywords used for thematic categorisation of autocomplete queries; Table S3: Top 50 most frequent autocomplete queries by language; Table S4: Representative example queries for each thematic category.

## Abbreviations

The following abbreviations are used in this manuscript:

BDBV	Bundibugyo ebolavirus
BVD	Bundibugyo virus disease
CDC	Centers for Disease Control and Prevention
CFR	Case fatality rate
DRC	Democratic Republic of the Congo
EVD	Ebola virus disease
IFRC	International Federation of Red Cross and Red Crescent Societies
MSF	Médecins Sans Frontières (Doctors Without Borders)
PHEIC	Public Health Emergency of International Concern
PPE	Personal protective equipment
RSV	Relative search volume
rVSV-ZEBOV	Recombinant vesicular stomatitis virus–Zaire Ebola virus (Ervebo vaccine)
VPN	Virtual private network
WHO	World Health Organization
