# Supplementary material for: Public Information Demand During the 2026 Bundibugyo Virus Outbreak: A Dual Infodemiology Analysis Across Six Ebola Virus Disease Outbreaks
Source: Viruses. 2026 Sep 8;18(9):987. doi: 10.3390/v18090987 (PMC13611964; doi:10.3390/v18090987)
Supplement: Supplementary file 1 [file viruses-18-00987-s001.zip › viruses-4530509-supplementary.pdf]

Supplementary Materials

Public Information Demand and the Francophone Misinformation Divide During the 2026 Bundibugyo Virus Outbreak: A Dual Infodemiology Analysis Across Six Ebola Virus Disease Outbreaks

Hakkı Öztürk and Berrak Itır Aylı

Table S1. Complete list of seed terms used for Google Autocomplete query extraction.

Each seed term was systematically expanded with 26 alphabetical suffixes (a–z), yielding up to 270 query inputs per term. A total of 83 seed terms were used (47 English, 36 French).

#	**English Seed Term**	#	**French Seed Term**
1	ebola	1	ebola
2	ebola virus	2	virus ebola
3	ebola disease	3	maladie ebola
4	ebola outbreak	4	épidémie ebola
5	ebola epidemic	5	fièvre ebola
6	ebola symptoms	6	ebola symptômes
7	ebola transmission	7	ebola transmission
8	ebola spread	8	ebola propagation
9	ebola prevention	9	ebola prévention
10	ebola contagious	10	ebola contagieux
11	ebola how do you get	11	ebola comment attraper
12	ebola airborne	12	ebola traitement
13	ebola cure	13	ebola vaccin
14	ebola treatment	14	ebola guérison
15	ebola vaccine	15	ebola mortalité
16	ebola survival rate	16	ebola taux de mortalité
17	ebola mortality	17	ebola congo
18	ebola death rate	18	ebola rdc
19	ebola diagnosis	19	ebola ouganda
20	ebola test	20	ebola afrique
21	ebola africa	21	ebola bundibugyo
22	ebola congo	22	ebola 2026
23	ebola drc	23	ebola peur
24	ebola uganda	24	ebola complot
25	ebola west africa	25	ebola faux
26	ebola 2026	26	ebola mythe
27	ebola bundibugyo	27	ebola remède naturel
28	ebola fear	28	ebola prière
29	ebola conspiracy	29	ebola quarantaine
30	ebola fake	30	ebola voyage
31	ebola myth	31	ebola risque
32	ebola hoax	32	ebola saignement
33	ebola natural remedy	33	ebola incubation
34	ebola prayer	34	ebola guéri
35	ebola quarantine	35	ebola survivant
36	ebola travel	36	ebola enfants
37	ebola risk	37	ebola grossesse

38	ebola bleeding		
39	ebola incubation		
40	ebola recovery		
41	ebola survivor		
42	ebola children		
43	ebola pregnancy		
44	ebola healthcare worker		

Table S2. Classification keywords used for thematic categorisation of autocomplete queries.

Each query was processed through categories in the priority order shown (top to bottom). A query was assigned to the first category for which a keyword match was identified. Keywords are shown for both English and French.

**Category (Priority Order)**	**English Keywords**	**French Keywords**
Misinformation & Conspiracy	conspiracy, hoax, fake, myth, lab, man-made, bioweapon, bill gates, depopulation, 5g, illuminati, government, cover up, lie, truth, secret, scam, fraud, patent, not real, propaganda, false flag, zombie	complot, faux, mythe, laboratoire, créé, arme, mensonge, vérité, secret, propagande, inventé, n'existe pas, zombie, manipulation, gouvernement, conspiration
Traditional & Alternative Remedies	natural remedy, natural cure, herbal, home remedy, traditional, prayer, faith, spiritual, healer, witch doctor, garlic, onion, salt water, vitamin c, zinc, colloidal silver, essential oil, bleach, miracle, alternative, neem, bitter kola	remède naturel, plante, remède maison, traditionnel, prière, dieu, guérisseur, ail, oignon, eau salée, huile essentielle, miracle, tisane, tradipraticien
Transmission & Contagion	transmission, spread, contagious, how do you get, airborne, droplet, contact, bodily fluid, blood, saliva, sex, mosquito, water, food, animal, bat, monkey, bushmeat, incubation, person to person	transmission, propagation, contagieux, comment attraper, aéroporté, gouttelette, contact, sang, salive, sexuel, moustique, eau, animal, chauve-souris, viande de brousse, incubation
Symptoms & Clinical Presentation	symptom, sign, early sign, stage, bleeding, hemorrhag, fever, vomit, diarrhea, rash, pain, headache, fatigue, weakness, red eye, sore throat, muscle, abdominal	symptôme, signe, premier signe, saignement, hémorrag, fièvre, vomissement, diarrhée, éruption, douleur, mal de tête, fatigue, faiblesse, yeux
Treatment & Medical Care	treatment, cure, medicine, medication, drug, therapy, antiviral, inmazeb, zmapp, monoclonal, plasma, clinical trial, hospital, rehydration, supportive care, recovery, survival rate, curable	traitement, guérison, médicament, thérapie, antiviral, plasma, essai clinique, hôpital, réhydratation, soins, taux de survie, guérissable
Vaccine	vaccine, vaccination, immuniz, ervebo, zabdeno, merck, dose, side effect, efficacy, effective, mandatory, ring vaccination	vaccin, vaccination, immunis, ervebo, dose, effet secondaire, efficacité, obligatoire, vaccination en anneau
Prevention & Protection	prevent, prevention, protect, precaution, avoid, hand wash, hygiene, sanitiz, disinfect, ppe, glove, mask, isolation, quarantine, lockdown, border, screening, burial, safe burial, funeral	prévention, protéger, précaution, éviter, lavage des mains, hygiène, désinfect, gant, masque, isolement, quarantaine, confinement, frontière, enterrement, funérailles
Epidemiology & Outbreak Updates	outbreak, epidemic, pandemic, cases, death, death toll, mortality, fatality, case fatality, how many, statistics, update, news, today, current, 2014, 2018, 2025, 2026, map, where, origin, history, first case,	épidémie, pandémie, cas, nombre de cas, mort, décès, mortalité, statistique, mise à jour, nouvelles, aujourd'hui, carte, où, origine, premier cas, patient zéro, oms, urgence

	patient zero, who, cdc, pheic, emergency	
Travel & Risk Assessment	travel, fly, flight, airport, safe to travel, travel ban, travel advisory, risk, danger, am i at risk, should i worry, europe, usa, uk, asia, india, china, canada, australia, near me	voyage, avion, aéroport, sûr de voyager, interdiction de voyager, risque, danger, europe, france, belgique, canada
Psychosocial & Fear	fear, afraid, scared, panic, anxiety, worry, stress, mental health, ptsd, trauma, stigma, discriminat, coping, support, emotional, orphan, family, community	peur, effrayé, panique, anxiété, inquiet, stress, santé mentale, traumatisme, stigmat, discrimination, soutien, aide, orphelin, enfant, famille, communauté
Healthcare Workers & Response	healthcare worker, health worker, nurse, doctor, medical staff, frontline, msf, doctors without borders, red cross, humanitarian, aid worker, contact trac, surveillance	personnel soignant, agent de santé, infirmier, médecin, première ligne, msf, médecins sans frontières, croix rouge, humanitaire, recherche de contacts, surveillance
Bundibugyo-Specific (2026)	bundibugyo, bdbv, bundibugyo virus, ituri, mongbwalu, bunia, no vaccine bundibugyo, new ebola, new strain, different ebola, type of ebola, species	bundibugyo, virus bundibugyo, ituri, mongbwalu, bunia, pas de vaccin, nouveau ebola, nouvelle souche, différent ebola, type d'ebola, espèce

Table S3. Top 50 most frequent autocomplete queries by language.

Queries are ranked by frequency of appearance across seed terms and alphabetical expansions. Many queries appear with frequency 1, reflecting the breadth of the autocomplete algorithm's output rather than repeated retrieval of identical suggestions.

#	**English Query**	**n**	**French Query**	**n**
1	was ebola a global health emergency	1	virus zika grossesse	1
2	ebola	1	ebola	1
3	ebola virus	1	ebola transmission	1
4	ebola outbreak	1	ebola symptome	1
5	ebola symptoms	1	ebola virus	1
6	ebola transmission	1	ebola rdc	1
7	ebola vaccine	1	ebola congo	1
8	ebola news	1	ebola france	1
9	ebola kenya	1	virus debut grossesse	1
10	ebola death rate	1	virus dangereux grossesse	1
11	ebola 2026	1	bola de grossesse	1
12	ebola virus kids	1	virus cmv grossesse c est quoi	1
13	ebola in children	1	virus coxsackie grossesse	1
14	ebola for kids	1	virus chat grossesse	1
15	ebola billionaire boys club	1	virus cmv grossesse en arabe	1
16	does ebola affect children	1	virus cmv grossesse symptômes	1
17	ebola and children	1	virus b19 grossesse	1
18	ebola virus in children	1	virus bebe grossesse	1
19	ebola children	1	virus grossesse enfant	1
20	ebola virus survivor	1	virus grossesse parvovirus	1
21	survivor ebola tab	1	virus grossesse cmv	1
22	survivor of ebola	1	virus grossesse	1
23	surviving ebola nova	1	ebola grossesse	1
24	ebola survivor lyrics	1	virus zona enfant	1
25	are ebola survivors immune	1	ebola définition	1
26	do ebola survivors have immunity	1	ebola cas	1
27	has anyone survived ebola	1	ebola congo brazzaville	1
28	ebola definition	1	ebola contagion	1
29	ebola death	1	ebola cameroun	1
30	ebola disease	1	ebola contamination	1
31	ebola drc	1	ebola cause	1
32	ebola cases in uganda today	1	ebola congo 2026	1
33	ebola cases in india	1	ebola canada	1
34	ebola canada	1	ebola c'est quoi	1
35	ebola cure	1	ebola beni	1
36	ebola cases in uganda	1	ebola butembo	1
37	ebola causes	1	ebola bouton	1
38	ebola china	1	ebola bilan	1
39	ebola cdc	1	ebola brazzaville	1
40	ebola congo	1	ebola bunia	1
41	ebola cases	1	ebola epidemie 2026	1
42	ebola bats	1	ebola en ituri	1
43	ebola bbc	1	ebola en afrique	1

44	ebola fatality rate	1	ebola en france	1
45	ebola english	1	ebola epidemie	1
46	ebola eyes	1	ebola europe	1
47	ebola explained	1	ebola en rdc	1
48	ebola en mexico	1	ebola danger	1
49	ebola early symptoms	1	ebola dans le monde	1
50	ebola epidemic 2026	1	ebola disease	1

Table S4. Representative example queries for each thematic category.

Up to five example queries are shown per language per category, selected from the first retrieved suggestions for each category. Queries are presented in their original form as returned by the Google Autocomplete algorithm.

Category	English Examples	French Examples
Epidemiology & Outbreak Updates	ebola outbreak; ebola news; ebola death rate; ebola 2026; ebola africa outbreak	ebola origine; ebola actuellement; ebola afrique 2026; ebola congo 2026; ebola cas
Transmission & Contagion	ebola transmission; ebola airborne; ebola bats; ebola from what animal; ebola how does it spread	ebola transmission; ebola incubation; ebola incubation period; ebola kisangani; ebola mode de transmission
Prevention & Protection	ebola lockdown; ebola lockdown in uganda 2026; ebola lockdown in uganda; ebola prevention; ebola precautions	ebola prévention; ebola quarantaine; ebola quarantaine canada; ebola virus lockdown; virus ebola prévention
Treatment & Medical Care	ebola cure; ebola medicine; ebola river; ebola survival rate; ebola treatment	ebola traitement; ebola kivu; ebola nord kivu; ebola river; virus ebola traitement
Travel & Risk Assessment	ebola australia; ebola american doctor; ebola china; ebola canada; ebola declaration form india	ebola france; ebola au canada; ebola belgique; ebola canada; ebola danger
Symptoms & Clinical Presentation	ebola symptoms; ebola bleeding; ebola bleeding eyes; ebola disease symptoms; ebola early symptoms	ebola symptome; ebola hémorragie; ebola signe; ebola signe clinique; ebola virus symptome
Vaccine	ebola vaccine; ebola virus biosafety level; ebola virus has vaccine; ebola virus vaccine; ebola virus vaccine in india	ebola vaccin; ebola virus vaccin; ebola y a t il un vaccin; virus ebola vaccin; virus ebola y a t'il un vaccin
Misinformation & Conspiracy	ebola zombie; ebola zombie virus; ebola virus zombie; what is the deadliest ebola virus; ebola transmission on a plane	ebola italie; ebola lieu; ebola zombie; virus ebola italie; virus ebola milieu de vie
Bundibugyo-Specific (2026)	ebola bundibugyo; ebola new strain; ebola virus bundibugyo; ebola disease bundibugyo; ebola disease caused by bundibugyo virus	ebola bundibugyo; ebola bunia; ebola en ituri; ebola ituri; ebola nouvelle souche
Psychosocial & Fear	ebola virus belongs to which family; ebola virus family; ebola disease family; ebola test family guy; eu ebola support drc uganda	virus ebola famille; fievre virus enfant duree; fievre virus enfant; contagion.virus enfant; bundibugyo virus family
Traditional & Alternative Remedies	ebola prayer; prayer for ebola outbreak	virus ebola taille; virus protection email; prevention virus huile essentielle; virus protection nasal spray; virus prevention nasal spray
Healthcare Workers & Response	ebola doctor; ebola disease surveillance portal; ebola doctor africa; ebola congo doctor; ebola dr congo msf	ebola congo doctor; ebola congo msf; ebola rdc msf
